# Perceptions and Attitudes Toward Utilizing a Non‐Invasive Biomarker for Colorectal Cancer Screening: A Qualitative Study

**DOI:** 10.1002/cnr2.70140

**Published:** 2025-04-11

**Authors:** Choi Ying Chu, Junjie Huang, Jamie Jie Mei Liew, Apurva Sawhney, Xianjing Liu, Chaoying Zhong, Jianli Lin, Junjie Hang, Claire Chenwen Zhong, Jinqiu Yuan, Martin C. S. Wong

**Affiliations:** ^1^ The Jockey Club School of Public Health and Primary Care, Faculty of Medicine The Chinese University of Hong Kong Hong Kong China; ^2^ Centre for Health Education and Health Promotion, Faculty of Medicine The Chinese University of Hong Kong Hong Kong China; ^3^ Department of Radiology and Nuclear Medicine Erasmus MC University Medical Center Rotterdam the Netherlands; ^4^ Department of Electrical Engineering and Automation Guangdong Ocean University Zhanjiang Guangdong China; ^5^ Peking‐Tsinghua Center for Life Sciences, Academy for Advanced Interdisciplinary Studies Peking University Beijing China; ^6^ Cancer Hospital & Shenzhen Hospital, Chinese Academy of Medical Sciences and Peking Union Medical College Shenzhen Guangdong China; ^7^ Clinical Research Center, Big Data Center, The Seventh Affiliated Hospital Sun Yat‐Sen University Guangzhou Guangdong China

**Keywords:** biomarker, colorectal cancer, DNA test, perception, screening

## Abstract

**Background:**

Colorectal cancer (CRC) screening, effective for early detection of CRC, was recently implemented by the Hong Kong government using the quantitative Fecal Immunochemical Test (FIT). However, consistently low participation rates and heavy reliance on colonoscopy for CRC screening has created a substantial burden on the healthcare system.

**Aims:**

This study examined the feasibility, acceptability, and satisfaction of utilizing the novel non‐invasive biomarker test Colotect for early detection of CRC.

**Methods and Results:**

In‐depth, qualitative telephone interviews were conducted with 16 participants (≥ 50 years old) who were eligible for the government CRC screening program. Participants were recruited via community health centres in Hong Kong between July and August 2022. The Consolidated Framework for Implementation Research (CFIR) was used to prepare the interview guide exploring the perceptions, attitudes, and barriers to using Colotect. The data collected was categorized into eight themes corresponding to the CFIR interventional characteristics domain, including intervention sources, evidence strength & quality, relative advantage, adaptability, complexity, design quality and packaging, and cost.

**Conclusion:**

Overall, participants reflected positive attitudes and perceptions toward Colotect use, along with identifying difficulties associated with its use. Modifications to test kits were proposed corresponding to the issues identified. In summary, this study demonstrated the feasibility and acceptability of using Colotect in CRC screening program. Future studies should examine the applicability, acceptability, efficiency, and cost‐effectiveness of the test in real‐world settings.

## 1 Introduction

Colorectal cancer (CRC) is the third most common cancer worldwide [1, 2, 3]. In Hong Kong, however, it is the second most commonly occurring cancer and cancer leading to death [3, 4, 5]. According to the Cancer Registry of Hong Kong, the age‐standardized incidence rate per 100 000 persons was 47.8 and 29.2 for men and women respectively in 2019. In the same year, the age‐standardized death rates were 17.1 and 10.0 for men and women respectively per 100 000 persons. The incidence rate of CRC is correlated with age, especially among people aged ≥ 50 years old, contributing to > 90% of newly diagnosed cases in Hong Kong [2, 4]. Given the ageing population in Hong Kong, the national CRC incidence will likely increase, translating to greater healthcare expenditure. The estimated cost increase for colorectal polyps to early stages (I and II) of CRC is over US$10 000, with further cost increases from stage II to stage III–IV cancers; the initial year of care for stage IV CRC is estimated at US$45 115 [6, 7]. These costs predict a significant public health burden related to CRC morbidity, mortality, and healthcare utilization in the near future.

CRC screening programs are important for early detection of CRC for decreasing mortality rates, particularly in increased‐risk groups. In 2018, the Hong Kong government implemented a population‐based CRC screening program, providing Fecal Immunochemical Test (FIT) kits to those aged between 50 and 75 years old after consultation with their primary care doctor [3, 5, 8]. Similar screening programs employing FIT have been rolled out with varying success across several Asian countries including Japan, Taiwan, and Korea with participation rates ranging from 21%–63%; the European recommendation for a minimum participation rate is 45% to demonstrate effectiveness of a screening program [7, 9, 10, 11, 12]. However, a 2019 Department of Health survey revealed that Hong Kong had the lowest participation rate of other Asian countries: 18.6% [3]. Several studies reported perceived barriers such as misconceptions regarding the program, lack of trust in screening effectiveness, and hesitancy towards screening procedures [2].

Recent advances also indicate that FIT may no longer be the most appropriate choice for screening programs, given their limited sensitivity to CRC (79%) and low sensitivity to adenomatous polyps (6%–56%), which can be precursors to CRC [13]. Colotect, a product from the Beijing Genomics Institute (BGI), is a DNA methylation detection kit. Colotect has several differences with the FIT (Table 1). While FIT simply detects the presence of blood in stool, Colotect detects novel biomarkers, specifically three methylated genes Syndecan‐2, alcohol dehydrogenase iron containing 1, and protein phosphatase 2 regulatory subunit B'gamma in stool samples. Unlike FIT, Colotect yields high sensitivity and specificity (87% and 92% respectively) for CRC [15]. Both sample collection procedures are similar, and Colotect provides additional hygiene equipment such as disposable gloves. Crucially, Colotect removes the need for colonoscopies due to its high sensitivity to adenomatous polyps, while current CRC screening programs recommend that patients with positive FIT results undergo colonoscopy to detect any adenomatous polyps. Colonoscopy presents several risks due to its invasive nature; the procedure is expensive and can be medically unsuitable for patients [9].

**TABLE 1:** Comparison of Colotect and FIT (iFOBT).

	Colotect	FIT
Invasive or non‐invasive	Both are non‐invasive screening methods	
Interpretation of test result	Both have standard equipment used for test in laboratory	
Cost	/	Free of charge of eligible participants joining the government subsidized CRC screening programme
Setting for the screening test	Self‐sample collection at home	
Usage in Hong Kong	A potential novel biomarker for diagnosis of CRC	Use in Hong Kong organized CRC screening programme as a primary CRC screening test Based on test outcome, users are recommended to undergo colonoscopy
Substance detected	Detects human genes SDC2, ADHFE1, and PPP2R5C methylation in stool specimens	Detection presence of hidden, invisible blood in stool
Equipment provided in the kit/used during the screening tests	Stool sample storage tube (with 4 mL preservation buffer) Disposable gloves Sampling paper Sample collection spoon One sealed plastic bag	Two sample collecting tubes Two small, sealed plastic bags One large, sealed plastic bag
Number of stool sample collected	1	1–2
Suitable population	Suitable for patients who have poor compliance with colonoscopy or are unable to undergo colonoscopy for medical reasons	Asymptomatic population at average risk to have annual or biennial FIT [14]
Specific restriction/preparation before screening	No drug and diet restriction	
Equipment provided in the kit	Stool sample storage tube (with 4 mL preservation buffer) Disposable gloves Sampling paper Sample collection spoon Sticker One sealed plastic bag	Two sample collecting tubes with preservation buffer inside Two small, sealed plastic bags One large, sealed plastic bag
Equipment provided by user	Garbage bag (depends on users disposal method after sample collection)	Garbage bag/ Newspaper Disposable gloves (depends on users' preference) Adhesive tape (depends on users' preference)
Preparation before the test	Open the package and take out all the items.	Users need to prepare garbage bag or newspaper for collecting the stool sample by themselves.
	Raise the toilet lid and seat.	
	And place the sampling paper (T‐shape) on the toilet.	And place the garbage bag/newspaper on the toilet.
	Stick the adhesive tape on the edge of the toilet seat and keep the sampling paper a certain concave curve.	Try to keep the garbage bag/newspaper as a certain concave curve
	Lower the toilet seat and have a bowel movement. Be careful do not allow stool to be in contact with urine or toilet water, as it will affect the test result.	
Stool sample collection process	Unscrew the stool sample collecting tube and make sure the tube is straight.	Twist the lid of the stool sample collecting tube.
	Sample the stool at 3–5 locations with the collection spoon and take a flat spoon of sample	Scratch sampling probe across stool surface in all directions. Specimen will be enough when the grooved part of the sampling probe is completely filled up.
	Transfer the collected sample into the storage tube and push the head of the spoon into the tube. Tighten the cap of the tube.	Insert the sample probe back into the collecting tube and screw it tightly.
After sample collection	Mix it gently.	/
	Put the collecting tube into the original packing and seal it.	Put the collecting tube into the small plastic bag and seal it. Please the collected sample in a cool place.
	Dispose of the sample collection spoon and disposable gloves. The sampling paper is made of toilet flushable material which can be removed through toilet flushing or disposing of it in a garbage bag.	Dispose of the garbage bag/newspaper used for collecting stool
Repetition of the screening process	No repetition is needed.	Repeat the same steps within 4 days, to collect the second tool specimen. After two specimen samples are collected, place the collecting tubes in the large plastic bag.

This accurate, non‐invasive DNA test may be a potential alternative for early detection of colorectal adenoma and CRC in asymptomatic patients. However, these novel biomarkers have never been evaluated as part of a CRC screening program in Hong Kong. If proven feasible, this low‐cost and simple DNA test may contribute to a new screening surveillance strategy that detects adenoma, predicts adenoma recurrence, and minimizes the need for colonoscopy in CRC screening. The objective of this qualitative sub‐study is to evaluate the feasibility and acceptability of using the novel non‐invasive biomarker test Colotect in a CRC screening program.

## 2 Materials and Methods

### 2.1 Participants

Participants who had been recruited to the larger Colotect study, a quantitative evaluation of the effectiveness, were invited to take part in this qualitative sub‐study at least 1 week after the intervention (the use of Colotect) was administered. About 16 participants were recruited in July and August 2022. Participants provided informed consent prior to the interview process. In‐depth interviews ranging between 20 and 40 min in length were conducted over the phone. Interviews were recorded for transcription and analysis. A sample size of 16 is justified in this qualitative study because it can reach data saturation, allowing for in‐depth exploration of participants' experiences while being manageable within practical constraints like time and resources, and it is consistent with similar research that has used comparable sample sizes effectively.

### 2.2 Recruitment

Purposive sampling was used to identify potential participants from community centres in different districts including the Neighborhood Elderly Centres (NECs), District Elderly Care Centres (DECCs) and the Family Integrated Services Centres (FISCs) who were eligible for CRC screening as of the year of the program, according to their age and the program‐specific eligibility criteria for the larger Colotect study. Eligible participants were grouped according to their characteristics: aged 50–64 versus aged 65 and over, male versus female, and educational level at primary school level and below versus secondary school level and above.

### 2.3 Data Collection

The original version of the Consolidated Framework for Implementation Research (CFIR), as presented in the 2009 paper by Damschroder et al., was utilized to prepare a semi‐structured interview guide, primarily focusing on the intervention characteristics domain [16]. Open‐ended questions were designed to explore perceptions and attitudes towards the use of Colotect in the CRC screening program. Questions were mapped to all constructs within the interventional characteristics domain, including intervention source, evidence strength and quality, relative advantage, adaptability, complexity, design quality and packaging, and cost (Table 2). The interview guide included knowledge of Colotect and the CRC screening program, awareness of evidence about the effectiveness of Colotect, perceived benefits of Colotect, adaptability, availability, and useability of Colotect, perception of the packing, supporting materials and quality of Colotect, cost consideration of Colotect, potential areas of improvement for Colotect, and whether they would recommend Colotect to family members or friends. The interview guide was pilot‐tested with a sample of eligible participants, with adjustments made based on feedback. Each interview was conducted by a researcher with extensive experience in qualitative research methodology in public health.

**TABLE 2:** Interview guide mapped to CFIR constructs from the intervention characteristics domain.

CFIR construct	Short description	Topics mapped to constructs
Intervention source	Perception of key stakeholders about whether the intervention is externally or internally developed.	Knowledge and perceptions of the developer of Colotect
Evidence strength and quality	Stakeholders' perceptions of the quality and validity of evidence supporting the belief that the intervention will have desired outcomes.	Awareness of evidence around the effectiveness of Colotect; types of evidence that would be useful
Relative advantage	Stakeholders' perception of the advantage of implementing the intervention versus an alternative solution.	Perceptions of the benefits or drawbacks of using Colotect compared to previous experiences with other FIT kits
Adaptability	The degree to which an intervention can be adapted, tailored, refined, or reinvented to meet local needs.	Identification of core and adaptable components of Colotect
Complexity	Perceived difficulty of the intervention, reflected by duration, scope, radicalness, disruptiveness, centrality, and intricacy and number of steps required to implement.	Perceived difficulty of implementing Colotect based on usability
Design quality and packaging	Perceived excellence in how the intervention is bundled, presented, and assembled.	Attitudes towards the presentation of and suitability of instructions for using Colotect
Cost	Costs of the intervention and costs associated with implementing the intervention including investment, supply, and opportunity costs.	Cost as a barrier or facilitator to using Colotect; examination of cost thresholds

### 2.4 Data Analysis

Interviews were transcribed from audio‐recordings, which were independently checked by another research team member for accuracy, and translated into English (Data S1). All transcripts were coded deductively in NVivo (version 14) by two research team members. Any disagreements were resolved by discussion. Finalized themes and their associated codes were then mapped to the CFIR. To strengthen the study's reliability and validity, we employed an audit trail ensures transparency and reproducibility, peer debriefing enhances objectivity by providing critical feedback from colleagues, and prolonged engagement fosters deeper understanding and trust with participants.

## 3 Results

About 16 participants were interviewed (Table 3). Data saturation was achieved after six interviews, from which eight themes were identified.

**TABLE 3:** Participant characteristics.

No.	Age group	Gender	Educational level
Participant 1	50–64 years old	Male	Secondary or above
Participant 2	50–64 years old	Male	Secondary or above
Participant 3	50–64 years old	Female	Secondary or above
Participant 4	50–64 years old	Male	Secondary or above
Participant 5	≥ 65 years old	Male	Secondary or above
Participant 6	50–64 years old	Female	Secondary or above
Participant 7	50–64 years old	Male	Primary or below
Participant 8	50–64 years old	Female	Primary or below
Participant 9	50–64 years old	Female	Primary or below
Participant 10	≥ 65 years old	Male	Secondary or above
Participant 11	≥ 65 years old	Female	Secondary or above
Participant 12	≥ 65 years old	Female	Secondary or above
Participant 13	50–64 years old	Male	Secondary or above
Participant 14	50–64 years old	Male	Secondary or above
Participant 15	50–64 years old	Female	Primary or below
Participant 16	50–64 years old	Male	Secondary or above

### 3.1 Theme 1: Intervention Source

The majority of participants were not cognizant of the developer of the test kits.

I don't know about it, and I don't pay attention to it. (Participant 11)

The few participants that did recall the developer had obtained this information from the kit packaging, or during the consent process for initial recruitment to the Colotect study.

It is on the packaging of the kit. (Participant 2)

The CUHK (The Chinese University of Hong Kong) staff told me about it, but I didn't pay much attention to the packaging when I used the kit. Because the staff talked about it clearly. (Participant 1)

### 3.2 Theme 2: Evidence Strength and Quality

Most participants were unaware of any evidence supporting the effectiveness of the test kit, expressing a high level of trust in the developers of the kit.

It's just a matter of faith, I believe that the laboratories entrusted by the government should be OK. (Participant 2)

Information or statistics…Well, I really don't know much about such a thing. (Participant 9)

When asked about the type of information that would be helpful for understanding the evidence behind the kit, participants suggested that reviews from experts such as researchers or scientists would be sufficient. Participants highlighted a lack the expertise to evaluate the effectiveness of a kit on their own.

Because the test kit is given by the hospital, the hospital and the staff who gave me the test kit told me about it, I would believe what they said. I did not mean to check anything, I have no way to prove whether the test kit is effective or not. (Participant 14)

### 3.3 Theme 3: Relative Advantage

Participants shared their perceptions of the advantages and limitations of the test kit compared with other brands or methods of stool testing. Strong positive sentiments were demonstrated regarding the kit. Participants highlighted its user‐friendly, hygenic, and convenient design.

Convenient, and easy to use. Easy to operate … the design and use are better and more convenient. (Participant 2)

However, certain participants encountered issues with the kit equipment.

It was not easy to remove the sticker from the toilet board. (Participant 3)

For the collecting tube holding the feces, it is very easy to rub to the edge of the sample storage tube, it would be better if the spoon is thinner. (Participant 4)

Around 75% of the participants had previously used different brands of test kits. These participants unanimously preferred this test kit due to its user‐friendly nature, presence of personal protective equipment, the appropriate size of the equipment.

The size of the equipment of other brands is smaller than yours. Of course, when you put the excrement into the collecting tube, it is not convenient for the elderly … for those with hand and feet tremors. (Participant 1)

The only difference was that you provided disposable sampling paper and gloves, which were much cleaner and reduced trouble. (Participant 16)

Participants who had not previously used test kits had experienced CRC screening through the hospital, which provided their own tools and materials for collecting stool samples.

Those hospitals did not mention that they can provide the test kits, they just gave you a sample bottle and let you collect samples at home. (Participant 2)

Most interviewees would recommend this test kit to their family members/friends due to various reasons, such as convenience, ease to handle, good quality, and hygienic design.

Yes, I would recommend it to friends or family members if they need it … Because I have used it before, I have experience … The whole procedure, quality, yes, the whole process. (Participant 1)

Yes, if you give me a choice, I will choose this one … Yes, it is more hygienic … I have used it before, and I think it is easy to use and convenient. (Participant 5)

It is convenient … Yes, the process is easy to do. (Participant 7)

### 3.4 Theme 4: Adaptability

Participants suggested aspects in which the test kit could be tailored and adapted to local needs.

### 3.5 Adaptable Elements of the Kit

The majority of insights focused on improving the accessibility of the kit. Participants proposed larger equipment, clearer graphics, instructions with larger font and in both English and Chinese languages.

I think it would be more convenient to use if the size of the sample storage tube is larger … Especially for elderly people that may sometimes have hand tremors. (Participant 1)

If elderly people receive the screening test, it would be better with clearer pictures. (Participant 9)

The alphabets need to be a bigger size, people with presbyopia cannot read them clearly It is not easy to read them. (Participant 16)

The only bad thing is that there did not have Chinese translation. Our education level is not high so we can only look at the pictures, and we do not understand English well. (Participant 5)

### 3.6 Core Components of the Kit

Participants approved of the inclusion of personal protective equipment, as well as design elements of the kit that allowed the procedure to be hygienic such as long spoons and disposable paper.

Gloves are worth keeping because I do not have to worry about touching the feces with my hands. Even if the disposable sampling paper for holding the excrement has any problem, I can deal with it immediately with a pair of gloves, so I do not need to touch it with my hand …. The spoon is long, so I don't have to come into close contact with feces. (Participant 3)

### 3.7 Theme 5: Complexity

Participants affirmed that the test kit was simple and easy to use, as clear instructions and sufficient equipment were provided, and the procedure itself was not complicated.

I just followed the procedures of pictures in the instruction manual given by you and performed them in the same way. (Participant 1)

The steps are not complicated. (Participant 4)

The whole process for me was user‐friendly, and suitable for home use. (Participant 10)

A couple of participants suggested that the kit may be too complex to use for individuals with low literacy, and that certain design elements could be simplified further.

If the person does not know how to separate the spoon, there should have someone to explain to him first. If he cannot understand it, he may use his own method to pull out the spoon head. (Participant 11)

Elderly people, I guess will find it difficult to use. It cannot rule out that some elderly people are illiterate. (Participant 13)

Most participants focused on the availability of test kits for the elderly since children or teenagers do not require CRC screening.

I think the support design, which can support the excrement is good, even the elderly who are clumsy still can use it to support the stool, and they still have a way to collect the sample. (Participant 3)

It's OK, you taught them to use it, I think there are very clear instructions, I think it's OK. It's like I don't need to read the instructions when I open the test kit, I also understand how it works. If you talk about the elderly and children, I think children are too small that they do not know how to use them and need parents' assistance. If it is for the elderly, there would be no problem, just with a slower process. I think that is it. (Participant 9)

### 3.8 Theme 6: Design Quality and Packaging

Most participants expressed satisfaction with the presentation of the packaging. They considered the design professional, practical, and hygienic.

I think … yours is very professional and ‘pro’. If it is too fancy, some elders may not know how to use it. (Participant 1)

No, I think it's ok to use this packaging to wrap it up like this. It's all so simple and doesn't need to be too fancy. (Participant 13)

A strong positive attitude toward the assembly of the kit was demonstrated, highlighting the acceptability of several components of the kit. Hygienic packaging and high quality materials were the main factors affecting participants' use of the kit.

Individual packaging, everything is clearly separated, such as gloves … spoons … reagent is individually packaged, which is already good for a user. (Participant 5)

Spoons, the materials are quite good … the hardness is appropriate, and it is easy to collect the sample. (Participant 13)

Several areas for improvement were proposed by participants regarding the design quality and range of materials provided in the kit.

I do not know about the texture of the sample storage tube … it feels like it is easy to crack, it is easy to crack when pressed so it is not good. (Participant 7)

After collecting the sample, can use higher quality sealed bags, for placing the sample, I think it will be better. (Participant 16)

Then the design of the removable spoon head is not needed, this design is not necessary. (Participant 2)

It would be better if the disposable sampling paper was easy to tear. (Participant 16)

Participants indicated that the availability of kits among elderly people depended on their health status.

The elderly can use it, as long as they are not mentally handicapped, or have dementia can use the kit. They are the same as ordinary people and can be used as long as they do not have any mental illnesses. (Participant 8)

### 3.9 Theme 7: Cost

Certain participants cited that cost may be a barrier depending on the financial circumstances of each individual, expressing that many would not use the kit if the cost was high.

So, considering individual abilities, for some people, probably a small amount of money, but for them, it is worth a penny. If we need to pay for the test kit, the cost is relatively high. (Participant 3)

If it is too expensive, it is not good. I think many people will not support it. (Participant 8)

Participants indicated that the acceptable price of a kit was tens of Hong Kong dollars.

It shouldn't be higher than HK$30. (Participant 14)

Around HK$20–30. (Participant 7)

If it is at our own expense, I think it should be under HK$100. (Participant 1)

Other participants stated that factors such as necessity and convenience may be prioritised over price of the kit.

No, no hindrance, because it is necessary to use. (Participant 1)

Of course, convenience will be considered first. The second is the price and the third is the credibility of the company. (Participant 13)

### 3.10 Theme 8: Others

Interviewees were asked about additional comments and suggestions they might have, and whether they would recommend the test kit to their family members or friends.

### 3.11 Additional Comments/Suggestions

Several interviewees had additional suggestions. One of them proposed that the test kit needed more promotion with its acceptability increased among people.

If this product is good, it should have more promotion, which means people of different ages can use it when they are needed, it would be better if more people can accept it. (Participant 1)

## 4 Discussion

This qualitative study explored the perceptions and attitudes of users regarding the novel DNA test CRC screening in Hong Kong. By focusing on the user experience, the study identified key barriers and facilitators to the acceptance and utilization of Colotect, providing valuable insights into how this test can be integrated into the CRC screening program from the user's perspective.

Generally, participants were knowledgeable about the current CRC screening program in Hong Kong, including risk factors for CRC and various methods of screening. Previous literature has shown that individuals' decisions to participate in CRC screening are affected by adequate knowledge on symptoms, risk factors, CRC screening approaches, and promotion of CRC screening [4, 17]. However, most study participants had not taken part in the CRC screening program. This low engagement is reflected in the population, as only 12.8% of Hong Kong citizens aged 50–75 have participated in the government CRC screening program, far below recommended guidelines [12, 18, 19]. While participants are aware of the purpose of CRC screening, other factors may affect whether eligible individuals choose to participate in the CRC screening program in Hong Kong.

Regarding perceptions toward Colotect itself, we identified a consistent lack of concern regarding the developer of Colotect and any evidence around the effectiveness of Colotect. Participants felt unqualified to assess the effectiveness of the kit, and had a high degree of trust in screening kits endorsed by trusted institutions such as the government, laboratories, or universities. This high level of faith in respected authorities aligns with research demonstrating that doctor's recommendations were associated with increased CRC screening participation, suggesting that recommendations by primary care doctors may result in the eligible target population being more likely to participate in screening [3, 4, 20, 21].

Psychological perceptions toward CRC screening has been shown to affect individuals' screening choices and participation [3, 5, 9, 22]. Overall, participants expressed strong support towards the use of Colotect as several core and adaptable components made the kit convenient, user‐friendly, high quality, and hygienic. This study suggests that convenience and user‐friendliness of the CRC screening test may play a role in affecting acceptance and intention to use Colotect. Participants noted the high quality of materials used to assemble Colotect. Unlike with Colotect, a recent review found that protective equipment is not provided in most CRC screening kits, despite previous research showing individuals were less likely to use stool collection kits due to the lack of hygiene [17].

Potential barriers to using Colotect revolved around the accessibility of instructions and materials. Generally, literacy level as a barrier to CRC screening remains inconclusive, but participants highlighted that individuals unable to understand English, such as the elderly, or those with low literacy would not be able to understand Colotect instructions [22, 23]. Simple instructions with large font have been shown to facilitate CRC screening participation, aligning with participants' reflections [24]. Participants also emphasized the value of additional resources to improve the accessibility of Colotect. Condensing comprehensive steps into educational videos akin to those currently available through the CRC screening program is preferable to text and should be considered along with enquiry hotlines, and mail delivery options to alleviate time‐constraints [9, 17, 20, 25]. Design modifications to the test kit such as a larger sample container or different stool collection tool were suggested by participants to increase hygiene and accessibility. Previous studies have identified similar design aspects of CRC screening kits that affected individuals' willingness to undergo screening [24, 26].

The cost of CRC screening kits was a key concern for participants, replicating previous studies that identified cost as a major barrier to CRC screening [9]. Although colonoscopy is considered a more effective approach than CRC screening kits for adenoma, advanced neoplasia, and early stages of CRC, its high cost in Hong Kong remains a significant barrier, contributing to colonoscopy as a less feasible option than screening kits [27, 28]. Indeed, participants outlined an acceptable cost range for Colotect as less than HKD 30 (around USD 4) for them to consider using the screening kit. As the FIT kit is currently free for eligible individuals through the Hong Kong CRC screening program, the use of Colotect in the program as an alternative may also be subsidized.

### 4.1 Strengths and Limitations

This qualitative sub‐study explored insights from a diverse range of participants with similar demographics to the end‐users of Colotect, if implemented in the CRC screening program in Hong Kong. However, the sample size was 16, and a larger size may be needed to obtain more generalizable findings. Certain participants with prior experience undertaking CRC screening or using test kits may have been more comfortable sharing their opinions around feasibility and acceptability compared to participants who did not. A quarter of participants had not used other stool collection kits previously, so the relative advantages or disadvantages of Colotect may not have been covered adequately. Participants had also been recruited from a larger quantitative study evaluating the effectiveness of Colotect where they had trialled the intervention 1 week prior, potentially leading to availability and social desirability biases. While we used CFIR, a robust and widely accepted framework, to evaluate several aspects of implementation, the barriers and facilitators identified solely focused on the interventional characteristics domain of the CFIR. Further research should explore individual‐level factors with other end‐users such as health professionals, as well as system‐level factors through the inner and outer settings domains.

## 5 Conclusion

This study suggests that Colotect is a feasible and acceptable option for CRC screening that could be implemented in the CRC screening program in Hong Kong. Through the interventional characteristics domain of the CFIR, we identified several barriers and facilitators to participant use. As the intervention itself has been deemed feasible and acceptable by participants who would be using the kit, future research should investigate perceptions of a wider range of end‐users around Colotect replacing FIT as the primary screening approach in the current CRC screening program in Hong Kong, covering the remaining domains in the CFIR to determine system‐level barriers and facilitators to implementing Colotect. A deeper understanding of implementing Colotect in the national CRC screening program may facilitate greater participation rates, ultimately improving health outcomes for all and reduce the public health burden on the health system in Hong Kong.

## Author Contributions

**Choi Ying Chu:** data curation (equal), formal analysis (equal), writing – original draft (equal). **Junjie Huang:** conceptualization (equal), supervision (equal), writing – original draft (equal). **Jamie Jie Mei Liew:** writing – original draft (equal). **Apurva Sawhney:** writing – original draft (equal). **Xianjing Liu:** writing – review and editing (equal). **Chaoying Zhong:** writing – review and editing (equal). **Jianli Lin:** writing – review and editing (equal). **Junjie Hang:** writing – review and editing (equal). **Claire Chenwen Zhong:** supervision (equal), writing – review and editing (equal). **Martin C. S. Wong:** conceptualization (equal), supervision (equal), writing – review and editing (equal).

## Ethics Statement

Survey and Behavioural Research Ethics (No. SBRE‐22‐0454), The Chinese University of Hong Kong, Hong Kong SAR.

## Consent

Written informed consent was obtained from the individual(s).

## Conflicts of Interest

The authors declare no conflicts of interest.

## Supporting information


Data S1.

## Data Availability

The datasets used and/or analysed during the current study are available from the corresponding author on reasonable request.
