# Supplementary material for: Perceptions and Attitudes Toward Utilizing a Non‐Invasive Biomarker for Colorectal Cancer Screening: A Qualitative Study
Source: Cancer Rep (Hoboken). 2025 Apr 11;8(4):e70140. doi: 10.1002/cnr2.70140 (PMC11986842; doi:10.1002/cnr2.70140)
Supplement: Supplementary file 1 — Data S1. [file CNR2-8-e70140-s001.docx]

**Supplementary material 1: Transcription of codes (translated from Chinese)**

**Theme 1: Intervention source**

**Description of the procedure or steps that have been tried**

*I just followed the procedures of pictures in the instruction manual given by you and performed them in the same way. (Participant 1)*

*The product procedure is based on your instructions. After opening the left wrapping paper, you will see a piece of paper, and then there is a sticker. The sticker is attached to the toilet and fixed. It's like that concave position, because it can hold feces. Then stick the piece of paper to the back, just below the anus, so that when you urinate in the front, the paper will not get wet. When I was doing this, there was always a paper towel spread under the toilet, even if trouble would pop up. Do you understand what I meant? (Participant 4)*

**Knowledge about the developer of the test kit**

*BGI. (Participant 16)*

*It’s not easy to remember, it’s really easy to remember. (Participant 5)*

*I don't know about it, and I don't pay attention to it. (Participant 11)*

**Ways to know about the developer of the test kit**

*It is on the packaging of the kit. (Participant 2)*

*The CUHK staff told me about it, but I didn’t pay much attention to the packaging when I used the kit. Because the staff talked about it clearly. (Participant 1)*

*I didn't pay attention to the packaging..., I'm sorry to try it out. (Participant 9)*

**Knowledge about the usage of the test kit**

*Since it is a colorectal cancer test, I would like to try the next function..., or it is not convenient to use (Participant 3)*

*Let's do a stool test, test some intestines, and screen for colorectal cancer. (Participant 15)*

*Let you do this colorectal cancer screening test. (Participant 16)*

**Ways to know about the usage of the test kit**

*That day in [Prince of Wales Hospital], a colleague of yours asked me whether I should participate in this experiment, and if I did, he would let me know about the study. (Participant 16)*

*Your colleagues already knew this when they told me. I read the instructions afterwards, which is why I know how to use it. (Participant 2)*

*Because I have used other brands before and they all have such descriptions, so I probably know this brand very well. (Participant 1)*

*In fact, colorectal cancer has been eliminated recently. Televisions sell advertisements all the time, and those over 50 years old are already eligible for colorectal cancer screening. (Participant 6)*

**Knowledge about CRC screening program**

*I know it is a pilot project. I think if people know if there are polyps in the large intestine, they can prevent it from becoming cancer as soon as possible. (Participant 11)*

*How much do you know about this plan? It's probably because you want to identify the age group you need for screening. Will you identify some high-risk individuals? For example, if there are many cancers in the family, how many similar objects are there? (Participant 9)*

*What I know is that there is a high chance of developing colorectal cancer by the age of 50. The government has some propaganda in public hospitals, and you can voluntarily participate. Pick the center left and you can have a stool test to try to prevent colorectal cancer. (Participant 14)*

*One day in 2018, I had a lot of blood in my stool. I went to see a doctor at the Health Department of CUHK. I talked to the doctor. The doctor then told me to see a specialist. He told me that I went to Prince of Wales Hospital. I arranged to go to the junior college and then he quickly received the one that was originally expired. Then he sent a letter saying that there would be a screening at home soon, and asked me to join the program. I joined a program and he helped me take care of my intestines very quickly. (Participant 6)*

*It's convenient to check the stool. It's just a stool test. [For colonoscopy] if you have to go up and take care of the intestines, it will be more laborious. You also have to eat a lot of food. You will eat until you become sick, and it will become clean. I don't feel clean about this. There are two kinds of laxatives. One is for those who already have polyps. I feel better if I take it. So I don't have [the polyps]. So the doctor [prescribed the wrong type of laxatives]. I ate so much and got sick so many times. I don’t have any confidence in [colonoscopy]. Well, I don’t know if it’s clear or not.* *(Participant 12)*

**Ways to know about CRC screening program**

*When I go to medical clinics or public hospitals, I see advertisements. (Participant 1)*

*There is propaganda all around, on TV, radio, newspapers and tape recorders. (Participant 2)*

*The media on the Internet have free newspapers online. (Participant 4)*

*On TV, the news talked about it. Some friends also told me about the pamphlets I saw in the hospital, and some colleagues participated. (Participant 14)*

**Theme 2: Evidence Strength & Quality**

**Type of information or evidence for the awareness of the effectiveness of a test kit**

*It's just a matter of trust. If the laboratory is entrusted by the government, it should be OK. (Participant 2)*

*Let's talk about it, something like this will be launched on the market, and if it is considered good, it will be launched on the market for people to use. (Participant 8)*

*Information or statistics…Well, I really don't know much about such a thing. (Participant 9)*

*That's okay, I know there are some numbers and stuff like that. (Participant 14)*

**Supporting evidence to help believing about the effectiveness of a test kit**

*To us citizens, we, the citizens, are different from the academics and scientists who have tried and tested them. In short, they can be trusted to be launched by the government or the relevant laboratories or the Chinese University of China. They have checked and proved it. These detection systems are effective. If it doesn't work, the public will be asked to verify whether it is effective. (Participant 2)*

*I don't have any special considerations about this. Because the test kit is given by the hospital, the hospital and the staff who gave me the test kit told me about it, I would believe what they said. I did not mean to check anything, I have no way to prove whether the test kit is effective or not. (Participant 14)*

*If there is such information, I will pay attention. If it is not done... there is no way, and I am not a professional and I don't know. (Participant 16)*

**Theme 3: Relative Advantage**

**Opinion about the test kit after use**

*I think this product is very positive, it’s OK. (Participant 1)*

*Convenient, and easy to use. Easy to operate… the design and use are better and more convenient. (Participant 2)*

*The process went smoothly, the stickers were not unstable, and the weight was not too heavy, so the whole system was very stable. (Participant 4)*

*I think the reagents are pretty complete, especially having gloves is very considerate. At least it's more convenient when doing that. You don't have to wash your hands before doing it. If something happens [and you lose the gloves], you just have to pick up some dirty things with your hands. (Participant 13)*

*It was not easy to remove the sticker from the toilet board. (Participant 3)*

*For the collecting tube holding the feces, it is very easy to rub to the edge of the sample storage tube, it would be better if the spoon is thinner. (Participant 4)*

*The spoon had to be placed in the bottle. However, I was not sure how to use the spoon. (Participant 8)*

*The only bad thing is that there did not have Chinese translation. Our education level is not high so we can only look at the pictures, and we do not understand English well. (Participant 5)*

**Comparison with other test kits**

*The size of the equipment of other brands is smaller than yours. Of course, when you put the excrement into the collecting tube, it is not convenient for the elderly… for those with hand and feet tremors. (Participant 1)*

*Since [this kit was] more comprehensive, since there are many accessories provided, it becomes easier to use. (Participant 3)*

*It’s just more convenient than what I did before. (Participant 10)*

*This [kit] will be more convenient because it has a pocket designed for [your stool], so you don't have to think of ways to catch it. (Participant 11)*

*[In previous kits] there are not many accessories, no gloves, no pad. (Participant 15)*

*The same thing is that both times I have a reagent bottle and put my feces for testing. The only difference was that you provided disposable sampling paper and gloves, which were much cleaner and reduced trouble. (Participant 16)*

*If you really have to choose to use [this kit], at least [this] packaging will be more considerate. (Participant 13)*

*Those hospitals did not mention that they can provide the test kits, they just gave you a sample bottle and let you collect samples at home. (Participant 2)*

**Recommendation to friends/ family**

*Yes, I would recommend it to friends or family members if they need it…Because I have used it before, I have experience…The whole procedure, quality, yes, the whole process. (Participant 1)*

*Yes, if you give me a choice, I will choose this one…Yes, it is more hygienic...I have used it before, and I think it is easy to use and convenient. (Participant 5)*

*It is convenient…Yes, the process is easy to do. (Participant 7)*

**Theme 4: Adaptability**

**Effectiveness and convenience of the test kit**

*Ordinary citizens in our area need to know more [about the kit’s effectiveness]. (Participant 2)*

*I have no way of knowing [about the kit’s effectiveness], it's all because I trust you. (Participant 3)*

*[In terms of the kit’s] convenience, all the tools you need to use, such as these gloves and samples, are all ready and convenient. (Participant 2)*

*Conveniently, [the kit] comes with everything, including a spoon and gloves. (Participant 6)*

*Everything can be found conveniently during the process. (Participant 16)*

**Changes or alterations suggested for the test kit**

*I think it would be more convenient to use if the size of the sample storage tube is larger… Especially for elderly people that may sometimes have hand tremors. (Participant 1)*

*If the mouth of your bottle is a few millimeters wider, it will be more convenient. (Participant 13)*

*If elderly people receive the screening test, it would be better with clearer pictures. (Participant 9)*

*Step 1, step 2, and so on make it easier for [users] to meet and follow the sequence. (Participant 16)*

*It seems that it would be better if the D picture is changed to a big one. It seems that the Wu series is too aggressive, because I remember. . . I posted it and didn't do any special research for a week. I saw something, so in my impression, their pictures are too big, so if it's possible for the elderly, their sizes are better, that is, they are relatively large. (Participant 3)*

*Write it big, I can't see it, [I’m an] old man. (Participant 12)*

*The alphabets need to be a bigger size, the presbyopia cannot read them clearly. It is not easy to read them. (Participant 16)*

*But the only bad thing is that there is no Chinese translation. So our level is not that high, we can only look at pictures, so we don't understand your English very well. If you really use this set of things today, it would be better to have some Chinese. (Participant 5)*

*A Chinese manual is required. (Participant 15)*

*Then the design of the removable spoon head is not needed, this design is not necessary. (Participant 2)*

*It would be better if the disposable sampling paper was easy to tear, because I have to [try to] tear it open a few times. (Participant 16)*

**Parts of the test kit that should be kept**

*Gloves are worth keeping because I do not have to worry about touching the feces with my hands. Even if the disposable sampling paper for holding the excrement has any problem, I can deal with it immediately with a pair of gloves, so I do not need to touch it with my hand…The spoon is long, so I don't have to come into close contact with feces. (Participant 3)*

*How good is it to use double-sided tape to stick on toilet boards? I think they are all suitable. (Participant 10)*

*Every piece of paper is good, it has a nice design, and it’s not too big for washing. Every piece of paper is so easy to use. (Participant 12)*

**Theme 5: Complexity**

**User-friendliness of the test kit**

*It's not complicated, it's acceptable. (Participant 1)*

*Nothing complicated. Because I know how to use it as I get older. (Participant 5)*

*It's all easy and easy to use. (Participant 8)*

*You will understand at a glance that it does not appear in newspapers. Unless the user has never tried it before, it is generally better than before. (Participant 9)*

*The whole process for me was user-friendly, and suitable for home use. (Participant 10)*

*In fact, most of the instructions are clear, there are enough accessories, and it's easy to use. (Participant 16)*

*If the person does not know how to separate the spoon, there should have someone to explain to him first. If he cannot understand it, he may use his own method to pull out the spoon head. (Participant 11)*

*The steps are not complicated. (Participant 4)*

*It's easy just follow the instructions. (Participant 6)*

*Elderly people, I guess will find it difficult to use. It cannot rule out that some elderly people are illiterate. (Participant 13)*

**Theme 6: Design Quality & Packaging**

**Perception of the packaging of the test kit**

*I think everything about [the kit] is ok, yours is very professional and ‘pro’. If it is too fancy, some elders may not know how to use it. (Participant 1)*

*No, I think it’s ok to use this packaging to wrap it up like this. It's all so simple and doesn't need to be too fancy. (Participant 13)*

*He has a paper bag to put in to dry, even if it is sealed and dried, it is generally OK, and it is more hygienic. (Participant 8)*

*Everything is neat and tidy, just right. Just pay attention to a few things, such as double-sided tape on toilet seats, and it will be better. (Participant 2)*

**Supporting materials of the test kit**

*It's all enough, all clear. (Participant 1)*

*All easy to understand. It would be nice if there were explanations in Chinese, because I found them all in English. (Participant 11)*

*All in English, I just looked at pictures to identify them. (Participant 12)*

*Independent packaging is more important. (Participant 1)*

*[You should] directly instruct users on how to use your product, ensuring the steps. (Participant 2)*

*I have touched these materials myself, and I have never been confident that I can dispose of them correctly. (Participant 14)*

**Quality of the test kit**

*Individual packaging, everything is clearly separated, such as gloves… spoons… reagent is individually packaged, which is already good for a user. (Participant 5)*

*The gloves are OK, but they are not very thin, even though they look as thin as a plastic bag. (Participant 6)*

*The paper quality is pretty good and tough. It won't rot so easily. (Participant 9)*

*Spoons, the materials are quite good… the hardness is appropriate, and it is easy to collect the sample. (Participant 13)*

*That sticker? Yes, it's sticky enough. (Participant 14)*

*I do not know about the texture of the sample storage tube… it feels like it is easy to crack, it is easy to crack when pressed so it is not good. (Participant 7)*

*After collecting the sample, can use higher quality sealed bags, for placing the sample, I think it will be better. (Participant 16)*

**Theme 7: Cost**

**Cost attractiveness of the test kit**

*So, considering individual abilities, for some people, probably a small amount of money, but for them, it is worth a penny. If we need to pay for the test kit, the cost is relatively high. (Participant 3)*

*It's at your own expense. Let's see if it's too expensive. If it is too expensive, it is not good. I think many people will not support it. (Participant 8)*

*It’s affordable for me. But you may have financial difficulties. If you want to take it frequently and it is too expensive, then it is not affordable. (Participant 16)*

*It shouldn’t be higher than HK$30. (Participant 14)*

*Around HK$20-30. (Participant 7)*

*If it is at our own expense, I think it should be under HK$100. (Participant 1)*

*No, no hindrance, because it is necessary to use. (Participant 1)*

*No, I haven't thought about what [price] to consider. Whatever [the price], you have to use it. (Participant 7)*

*As for the cost, it is not important to me, so I don’t care. (Participant 10)*

*It's easy to use. The most important thing for me is that all the samples are easy to use. (Participant 2)*

*Convenience comes first. (Participant 7)*

*Of course, convenience will be considered first. The second is the price and the third is the credibility of the company. (Participant 13)*

**Theme 8: Others**

**Additional comments/ suggestions**

*If this product is good, it should have more promotion, which means people of different ages can use it when they are needed, it would be better if more people can accept it. (Participant 1)*
